# **The Multifaceted Pathways Linking Populism to Ethnic Minority Health** Comment on "A Scoping Review of Populist Radical Right Parties’ Influence on Welfare Policy and its Implications for Population Health in Europe"

**DOI:** 10.34172/ijhpm.2020.154

**Published:** 2020-08-15

**Authors:** Karien Stronks, Charles Agyemang

**Affiliations:** Department of Public and Occupational Health, Amsterdam University Medical Centres, University of Amsterdam, Amsterdam, The Netherlands.

**Keywords:** Migrant Health, Ethnic Minority Health, Populism, Discrimination, Systems Thinking

## Abstract

Based on a scoping review of empirical studies accompanied by interviews with experts, Rinaldi and Bekker studied the impact of populist radical right (PRR) parties on access to welfare provisions – the latter standing proxy for population health and for health inequalities in particular. We argue that populism can impact on migrant and ethnic minority health in multiple ways, in addition to the welfare mechanism specified in that review. These include institutionalised discrimination affecting individuals’ positions in the social hierarchy, experiences of discrimination in interpersonal relationships, and a weakened legitimacy of health policies. Interdisciplinary teams that include public health scholars and political scientists should take up the challenge of understanding migrant and ethnic minority health from a systems perspective.

## Introduction

Many immigrant groups from low- and middle-income countries that have settled in Europe show higher risks of health problems than the host populations of their adopted countries.^1^ For example, the risk of type 2 diabetes is three to four times higher in immigrants with South Asian backgrounds, such as the South Asian Surinamese minority in the Netherlands. In addition, higher levels of depressive symptoms in immigrants have been reported in most countries in northern and western Europe. When immigrant populations are differentiated into specific ethnic groups, the pattern of greater health risks appears to hold for most groups originating in low- and middle-income countries, though prevalence rates differ between groups as well as between health conditions.

The common explanations for the emergence of ethnic inequalities in health are reductionist in nature: the unequal risks across ethnic groups are generally unravelled into separate components, such as biological processes or specific behaviours. For example, the higher risk of type 2 diabetes in people of South Asian background may be coupled to factors like a higher prevalence of obesity in these groups, and a higher prevalence of depression in multiple ethnic minority groups to greater exposure to discrimination. Reductionist explanations like these, however, fail to provide insights into the reasons behind the single explanations, such as the factors that shape feelings of discrimination. Nor do they shed light on how those components interact with other possible explanations, such as the impact of psychosocial stress on health in the presence (or absence) of obesity. There is a growing consensus among public health researchers that, if this field is to move forward, the complexities underlying population health and its distribution require a shift in research paradigm towards *systems thinking*.^2^ In systems thinking, inequalities in health are conceptualised as an outcome of an interconnected web of factors (such as behaviours or environmental factors), at many levels (eg, individual level, group level, country level), involving many actors (eg, government, social network, family). These elements interact with one another, generating effects that persist over time, whilst the elements themselves may be modified by changing circumstances. Such a shift towards systems thinking is imperative to do justice to the fact that health inequalities, as a phenomenon, arise from social forces – forces that drive the life chances of people in various groups, which in turn drive variations in population health. Such forces remain invisible when reductionist methods are applied. In a reductionist perspective, the assumption is that social forces manifest themselves as risk factors at the individual level, predisposing individuals to obesity. With reference to ethnic inequalities in health, a systems perspective should cover both the unique and rather stable attributes of the group, such as genetics and culture, as well as living conditions to which such groups are exposed in the host country, including working and housing conditions, social networks, discrimination, income and educational opportunities.^3^

## Political Factors as Components of the System That Produces Population Health

Many elements of the system that ‘produces’ population health are shaped by public policies. Since the latter, in their turn, are shaped by the governance of a country, a systems perspective to understand population health and inequalities should include political variables. It is in this respect that the scoping review by Chiara Rinaldi and Marleen Bekker^4^ contributes to a better understanding of health inequalities. Their scoping review focuses on a specific element of the political system – the rise of populist radical right (PRR) parties in Europe. Though it is not stated explicitly, they appear to focus on the health of ethnic minority and migrant populations as a particular outcome. We appreciate this choice, given that ‘migration policy’ is considered the core issue of the PRR parties, related to ‘nativism’ as one of the elements that characterises the ideology of PRR parties. Other characteristic elements as mentioned by Rinaldi and Bekker include a critique of the elites in combination with the claim to give ‘the pure people’ a voice, and authoritarianism. On the basis of a scoping review of empirical studies, accompanied by interviews with experts, Rinaldi and Bekker studied the impact of PRR parties on welfare policy, the latter being a proxy for population health and for health inequalities. A key message of their review is that participation by PRR parties in government is likely to have negative effects on access to welfare provisions and, as a consequence, might negatively affect the health of groups that have been excluded. That impact does not appear to be uniform across political systems, however. In tax-based healthcare systems, for instance, demands for the restriction of health services are apparently stronger than in insurance-based systems, at least as far as the public discourse is concerned (irrespective of actual policy measures).

## Multifaceted Causal Pathways That Link the Populist Radical Right to Ethnic Minority and Migrant Health

Rinaldi and Bekker substantiate their focus on welfare policies as a proxy for population health by arguing that ‘welfare chauvinism is the *most prominent channel* [italics ours] through which PRR parties could adversely affect population health and health equity in Europe, as welfare chauvinistic policies have the potential to directly affect access to welfare provisions for vulnerable (immigrant) groups’ (p. 8). We would like to argue that other channels or pathways might be at least of equal importance and should therefore be considered in future research.

A first such additional pathway is centred around socioeconomic status, including educational and occupational level. As indicated in the above quote, the focus of Rinaldi and Bekker’s review is on immediate access to welfare provisions. We would like to draw attention to a longer-lasting effect of limited accessibility, which derives from the position that individuals occupy in the social hierarchy. Many studies across Europe have indicated that the average lower socioeconomic status of immigrant groups in high-income countries accounts for part of their higher risk of adverse health conditions.^5^ Educational level plays a central role here, by shaping employment and occupational opportunities as well as income. Although the educational levels of ethnic minority people are rising across generations, there are indications that equality of educational opportunity has not yet been achieved for the offspring of immigrants. In countries, for example, where children transition from comprehensive primary school classes to specific secondary education tracks (levels), pupils of minority ethnic background with similar performance ratings have been shown to have lower chances of entering particular levels as compared with children of majority background.^6^ Such a pattern might reflect institutionalised discrimination – inequities embedded in policies or procedures of organisations – as the school transition system allows room for bias in track recommendations.^7^ Such bias is likely to be strengthened if a climate of nativism arises as propagated by PRR parties, whereby immigrants are framed as a collective threat to exacerbate collective insecurity.^8^ In addition, although PRR parties generally seem to favour quality education, they also show tendencies towards strengthening national identity through education and emphasising free choice for parents for access to high-quality education.^9^ Both those factors might work to the disadvantage of school children from ethnic minority and migrant backgrounds and their parents. Institutionalised discrimination might also play a role in the ways that educational level shapes opportunities for employment. First-generation migrants have been shown to be more likely to have jobs that are below their level of education.^10^ Also, discrimination in the labour market, such as in recruiting procedures, might be fuelled by PRR hostility towards migrants and minority groups.

Second, the rise of PRR parties could have effects on migrant and minority health through individual discrimination – unfair treatment from majority individuals on grounds of ethnic background. Discrimination in interpersonal relationships has been shown to play roles in the elevated risk of depression in ethnic minority populations, in higher physical health risks for conditions like obesity, and in behavioural risk factors such as smoking and alcohol consumption.^11^ This applies not only to migrants themselves but to their offspring as well.^12^ Given the political rhetoric of PRR parties, with its heavy focus on policies relating to immigrants, as well as PRR opposition to basic principles of group-specific rights for minorities, this might foment hostility towards those groups in the host population. That may well happen independently of whether PRR parties participate in government, given the overall tendency in European societies, in areas like mainstream journalism, to normalise and legitimise the political views and language of the radical right in public discourse.^13^

A third mechanism concerns the legitimacy of health policy, and particularly of policies aimed at preventing disease, such as vaccination and health promotion efforts. Preventive policies are crucial in tackling health inequalities, as health inequalities largely reflect disparities in the *incidence* of disease, rather than in adverse outcomes once people have developed an illness. Given the central role of behavioural factors like smoking and dietary habits as downstream determinants in the origin of health inequalities, a key focus for tackling inequalities is to increase the number of people that adhere to healthy behaviours. Public health warnings to avoid risks such as smoking or obesity might be less well received in a context where PRR parties are in power, particularly in view of the non-establishment rhetoric they employ. For the distrust in elites that characterises PRR parties appears to extend to scientific evidence and medical experts.^14^ A clear example is seen in the politics of mask-wearing in the fight against the coronavirus in the United States, where – as a consequence of that country’s bitterly partisan culture war politics – large numbers of people refuse to wear protective masks, in defiance of scientific evidence and expert advice.^15^ This may particularly apply to PRR parties that adhere to a neoliberal ideology. Should the emphasis on individual responsibility in neoliberal health policies get interpreted as a violation of personal autonomy, that might become a boomerang that undermines the legitimacy of those same health policies.^16^ That would jeopardise the effectiveness of the policies and thus impact upon population health, especially in groups where unhealthy behaviours are highly prevalent.

## Interdisciplinary Research Based on Systems Thinking as a Next Step

 We appreciate the recommendation by Rinaldi and Bekker for further empirical research on the public health consequences when PRR parties hold office. If such research is to effectively support health policies, we argue that it should employ a systems approach that embraces the complexity of the multifaceted pathways linking this political factor to public health. To achieve that, an interdisciplinary approach is imperative, whereby downstream and upstream factors influencing public health are studied simultaneously and scholars from multiple backgrounds are involved. In such an approach, conceptions from political theory as to how PRR parties affect policies would be enriched by public health notions on possible mechanisms linking political factors and health, like those we have proposed in this commentary. Political scientists, in their turn, would enrich public health notions with knowledge and expertise on political factors. That would enable us to look beyond individual, downstream determinants of health, which are key when it comes to healthifying peoples’ living conditions. We hope our commentary will serve as a source of inspiration for taking up this interdisciplinary challenge.

## Ethical issues

 Not applicable.

## Competing interests

 Authors declare that they have no competing interests.

## Authors’ contributions

 KS conceived and wrote the initial draft. CA provided critical review and revision of the manuscript.
